# Testing the Representational Deficit Hypothesis: From the Aspect of Chinese Learners’ Acquisition of Affixation ‘-*s*’ for Third Person Singular Verbs and Plural Nouns

**DOI:** 10.3389/fpsyg.2022.930504

**Published:** 2022-06-10

**Authors:** Ni Li, Lianrui Yang

**Affiliations:** College of Foreign Languages, Ocean University of China, Qingdao, China

**Keywords:** representational deficit hypothesis, third person singular marking, plural noun marking, written and spoken tasks, Chinese EFL learners

## Abstract

The persistent difficulty in making verbal inflections is commonly recognized for second language learners, especially for Chinese-speaking students. Researchers put forward different hypotheses to explain the problems in acquiring inflectional morphology. Among them, the representational deficit hypothesis deficit (RDH), advocated by Hawkins and Liszka, indicates that adult learners will fail to make inflectional morphology to interpret the corresponding syntactic feature if there is no counterpart system in their native language. In English, affix morpheme ‘*-s’* marks either third person singular (3SG) in the present tense or regular plural nouns. In contrast, Chinese is a language which lacks 3SG markings but presents the morpheme ‘*men*’ to reflect a plural feature for nouns with a human property. To test the applicability of the RDH in the domain of affix ‘*-s*’ for English learners of Chinese, the present study observed the morphological inflections of the third person singular and plural ‘*-s*’ in 33 Chinese EFL learners’ written and spoken production tasks. The results show that the participants distinguished between the inflectional morphology in regular plural and 3SG thematic verb markings, which was compatible with the RDH. Additionally, other phenomena related to 3SG and plural morphological inflections provided strands of evidence for the RDH, for instance, L2 exposure age, a prominent overuse of plural ‘*-s*’, and exceptional cases for more 3SG ‘*-s*’ markings in the written data. Except for the account of morphosyntactic processes in the RDH, other factors, such as input frequency, difficulty of paradigm uniformity, and acquisition order, were referred to as the way that the L2 learners’ acquisition of the morphological inflections was affected.

## Introduction

Second language (L2) adult learners are faced with a long-lasting problem of acquiring inflectional morphology. A majority of research has demonstrated that L2 learners present optional suppliance for regular past tense marking, and this phenomenon is especially robust for Chinese learners (Bayley, 1996). In a case study conducted by Lardiere (1998a,b, 2017), a Chinese immigrant named Patty failed to supply past tense inflections at a native-like level, even though she had lived in an English-speaking country for over two decades. Besides, the suppliance rates of third person singular marking with morpheme ‘*-s*’ were observed to be substantially lower than the suppliance rates of past tense ‘*-ed*’ in the production from Patty. Moreover, compared with L1 (first language) German and Japanese learners of L2 English, Chinese learners showed a stronger impairment in inflecting verbs with past tense features (Hawkins and Liszka, 2003). Therefore, the existence of the optionality of morphological inflections is generally acknowledged.

However, there is no unified explanation for the poor performance in presenting inflectional markings. The representational deficit hypothesis (RDH) explains that the prior linguistic knowledge in L1 will have an impact on second language acquisition (Hawkins and Liszka, 2003). Specifically, if the abstract features (i.e., person, number, and tense) are not instantiated by morphological inflections in L1, learners will have difficulties in presenting correct inflections to decipher the features in L2. On the other hand, Goad et al. (2003) and Goad and White (2006) hold a skeptical view of the RDH while support the prosodic transfer hypothesis (PTH). However, no matter the RDH or the PTH, the corresponding research put emphasis on the past tense marking, while the investigation on a worse inflection for 3SG marking is ignored.

To identify the validity of the RDH, this study focuses on the investigation of two under-researched homophonous morphemes: third person singular (3SG) for verbs and plural for nouns. Under these two circumstances, affix ‘*-s*’ is obligatorily required for regular markings. As for Chinese, there is no agreement marking for 3SG, whereas, morpheme ‘*men*’ can follow nouns involving a human property to present a plural feature. Hence, based on the RDH, Chinese students will show better performance in inflecting plural ‘*-s*’ than 3SG ‘*-s*’. In order to increase the application range of the data, the investigation is not only carried out on a spoken task but also on a written task.

## Literature Review

### Representational Deficit Hypothesis

Based on the minimalist program, Chomsky and Collins (2001) states that ‘language faculty has a number of universally fixed and invariant computational procedures, and provides a universal inventory of phonological, semantic and syntactic (formal) features from which lexical items can be assembled’. Syntax is one of the computational procedures, which assembles lexical items into expressions through a series of related operations (Merge, Agree and Move). Functional heads can be valued by merging with lexical items. For example, Nouns are specified as nouns by merging with the functional items with an N (noun) feature, while verbs are specified with a V (verb) feature. In this way, the specified lexical items are categorized with (un)interpretable features. Specifically, the syntactic expressions can be produced in speech which is then understandable for language learners, and this step is completed through morphological or phonological procedures. Semantic, morphological, and phonological procedures are universally invariant for all languages, while different languages may select different features to undergo the computational procedures. Biberauer (2019) clarifies that ‘the language-specific ‘content’ of what it means to “be” categories of different types, and also what features are grammaticalized’. For example, there are some syntactic features, such as *wh*-movement and case agreement (Katamba et al., 2011), which are selected in English but absent in Chinese. In terms of the interface between syntax and morphology discussed above, if students are not exposed to the target language at an early age, learners will have persistent difficulty in acquiring the L2 features when the features are inconsistent with their L1 system. Hawkins and Chan (1997) first described this phenomenon as the FFFH (the failed functional features hypothesis), and then referred to it as the RDH in 2003.

### Previous Studies Related to the RDH

Hawkins and Liszka (2003) pointed out that the optionality in verbal inflections was attributed to the lack of corresponding syntactic features in L1. In the study, the researchers put emphasis on the morphological inflections for past tense marking from the view of advanced L2 English learners of three different first languages: Chinese, Japanese, and German. In English, the syntactic feature of T (tense) [±past] is uninterpretable in language faculty, and therefore the morphological inflections must occur in order to decipher the syntactic features. For example, ‘*is, have, walk*’ can be morphologically inflected to ‘*was, had, walked*’ by checking the [+past] syntactic feature. Both Japanese and German have [±past] T feature with morphological inflections as English does. However, there is no [±past] feature on T in Chinese lexicon^1^, which can be exemplified in Sentence (1). The verb *‘zhu’/‘live’* still keeps the bare form in the context of past tense.

(1) John shi nian qian zhu zai Beijing
*John ten year ago live in Beijing.*
  ‘John lived in Beijing ten years ago’.

In the study of Hawkins and Liszka (2003), the participants were firstly asked to use the correct forms of the given verbs in a cloze test. In order to improve the validity of the task, two types of verbs were involved: real and invented verbs, which included regular and irregular inflections for each category of verbs. The results demonstrated that all three non-native groups were able to correctly inflect regular verbs, while they failed to produce correct inflected forms of irregular verbs. In regard to the oral production task, the data was collected through retelling a short story and describing a prior experience. The results presented that the Chinese group inflected regular and irregular verbs less than the Japanese and German groups. This was different from the results in the written task where all three groups showed similar scores in inflecting verbs. It was then implied that the Chinese students showed optionality in supplying inflected verbs due to the lack of [±past] on T (tense) in L1. Chinese learners failed to check the feature between T and lexicon in IL. Consequently, the absence of the [±past] syntactic feature in L1 is the reason why Chinese learners show deficiency in marking regular verbs with past tense forms, which is consistent with the RDH.

In regard to the study of Hawkins and Chan (1997), the acquisition of *wh*-movement in L2 English was tested with 147 L1 Chinese, 113 L1 French, and 30 English native speakers as a control group. The researchers divided Chinese and French into three groups with different proficiency levels respectively: elementary, intermediate, and advanced level. The movement of *wh*-operator is allowed in French and English but is absent in Chinese. The participants were required to do a GJT (grammatical judgment test). The results showed that the Chinese elementary group got the lowest accuracy scores, while the native control group achieved the highest. The scores of the French elementary group were higher than all three Chinese groups. Hence, the study proved the FFFH (also the RDH) which assumed that Chinese learners should show less accuracy than French subjects in each level group owing to the difficulty from the lack of the relevant functional feature-*wh*-movement in L1.

In brief, it is the parameterized syntactic features that play a significant role in the operations between lexicon and morphophonology in IL, for instance, [±past] T feature in the study of Hawkins and Liszka (2003) and *wh*-movement feature (1997). If the correlated syntactic feature is absent in L1 and is not activated in a crucial period (Tsimpli and Roussou (1991) postulate that the period should be early childhood), the L2 learners may suffer from permanent impairment of the morphosyntactic structures since they are unable to set new parameters in IL grammar. This statement serves as the basic framework for the RDH and FFFH.

### From Previous Studies to the Present Study

#### From Affix ‘*-ed*’ for Past Tense to ‘*-s*’ for 3SG Verbs

In light of the relation between syntax and morphology in Chinese learners of English, most studies mainly focus on verbal inflections of past tense forms, while just mentioned 3SG inflections in a couple of words (Goad et al., 2003; Hawkins and Liszka, 2003; Lardiere, 2017). Although both regular past tense and 3SG inflections are at the bottom of the hierarchy in second language acquisition (Zobl and Liceras, 1994), it was noted that learners show more deficiency in supplying ‘*-s’* for 3SG than ‘*-ed*’ for simple past tense. For example, in the research of Goad et al. (2003), twelve Chinese adults who had lived in Canada for a period ranging from six months to five years were invited to be participants. The results of the production task demonstrated that the suppliance rate of 3SG was 28.46% (57/201), which was much lower than 57.14% (16/28) for regular past tense verbs. According to Lardiere (2017), Patty only inflected 4.41% (3/68) of lexical main verbs for 3SG and inflected 5.8% (8/138) for past tense. In addition, although both studies referred to inflections of 3SG, the former put emphasis on the prosodic features and the latter involved the realization of surface morphology, rather than the impairment of syntactic features. In addition, there is a study from Hsieh (2009) which invited twenty Taiwanese teenagers aged 11–13 as the respondents, who had learned English as a second language for four to 7 years. In this research, although the participants omitted regular past tense marking ‘*-ed*’ (95% in 35/37) more than 3SG affix ‘*-s*’ (78% in 148/176), the inflected rate of 3SG was low. Briefly, at least from the existing studies, it seems that Chinese advanced learners have more difficulty in supplying morpheme ‘*-s*’ for 3SG than supplying affixal past tense marking.

To find out why L2 learners omitted the 3SG marker, Blom et al. (2012) carried out the experiment on fifteen participants with different native languages, that is six for Mandarin, two for Cantonese, one for mixed Mandarin and Cantonese, five for Spanish, and one for Romanian. Both Mandarin and Cantonese are isolating languages which lack tense and agreement inflections, while Spanish and Romanian are richly inflected languages. The data of the spontaneous communication task revealed that L1 backgrounds had an impact on the performance in inflecting 3SG ‘*-s*’, since the subjects with L1s of Cantonese and Mandarin tended to drop more plural ‘*-s*’ than the ones with inflecting L1s (Spanish and Romanian). A similar L1 influence in L2 verbal morphology can also be found in other research (McDonald, 2000; Paradis, 2011), where participants with a rich inflecting L1 system performed better than those without agreement inflections in L1s.

As can be seen from the above empirical studies, Chinese-speaking students frequently omit 3SG marking with the affix ‘*-s*’; however, few research put emphasis on the relation between the RDH and the low suppliance rates of inflected 3SG verbs, which offers a research gap for the present study.

#### From Copula ‘*be*’ to ‘*-s*’ for Plural Nouns

In L1 Chinese speakers, the high inflected rates of copula ‘*be*’ can be found in both 3SG and simple past tense contexts (Goad et al., 2003; Hawkins and Liszka, 2003; Hsieh, 2009; Blom et al., 2012; Lardiere, 2017). There are only three forms of the auxiliary ‘*be*’ in English, namely, *is, am, are*, and thus some researchers believe that L2 learners are able to supply the ‘*be*’ forms by rote. On the other hand, Hsieh (2009) states that the similarity of native language (Chinese) and the target language (English) may help learners produce inflected copula ‘*be*’. ‘*Shi*’ in Chinese can be put between the subject and the complement, which behaves similarly to auxiliary ‘*be*’ verb in English. This idea is also supported by Chan (2004), who indicates that the more similar L1 to L2 is, the more likely L1 is to facilitate second language acquisition. In this way, ‘*shi*’ in L1 can have a positive effect on the acquisition of copula ‘*be*’ in L2. However, even if ‘*shi*’ in Chinese functions similarly with ‘*be*’ in English, there is no morphological marking of ‘*shi*’ for any agreement infections (Lee and Huang, 2004). Therefore, in the light of the RDH, it should be cautious to simply regard the high inflected rates of ‘*be*’ as the result of L1 transfer from ‘*shi*’.

Turning to the acquisition of the plural morpheme ‘*-s*’, L1 Chinese students also have the problem in supplying the relevant morphological inflections (Jia, 2003). The researchers conducted a five-year longitudinal study on ten Chinese children who immigrated to New York City between the ages of five to sixteen. After having been exposed to English for five years, only seven of the participants reached a high level in marking a plural feature. Among them, five were from a younger group and the other two were aged 12 and 15. The conclusion was then made that the initial age of exposure to L2 may affect the acquisition of the plural morpheme ‘*-s*’ in Chinese learners. This statement is partially related with the RDH, since an earlier exposure may be corresponded to ‘the crucial period’ in the RDH. However, the RDH focuses on the lack of a syntactic feature in L1 while this study ascribed the failure of supplying enough plural forms to neurobiological-related influences, such as cognitive and social contexts. According to Rullmann and You (2006), Chinese is not a language without number morphology. The morpheme ‘*men*’ can follow a human object to make a plural marking, such as ‘*pengyou-men*’ for ‘*friends*’ (Li, 1999). More detailed information about ‘*men*’ will be discussed in “Plural Marking in Chinese (‘men’ for Nouns With Human Property).” As such, compared with ‘*shi*’, ‘*-men*’ is more relevant to morphological inflections. However, there was few research associated the deficiency in acquiring plural morpheme *‘-s*’ with the RDH.

### Linguistic Background

#### Third Person Singular Marking

According to Katamba et al. (2011), number (singular/plural) and person (first/s/third) agreement is quite common in English. As for third person singular subjects in the present simple tense, thematic verbs must be suffixed with ‘*-s*’ to mark the 3SG feature due to the subject–verb agreement. For example, *‘walks’* has the feature of [verb, +finite, third person, singular, −past], while ‘*walked*’ conveys the feature of [verb, +finite, +past]. Turning to the morphological inflection of auxiliary verbs: *be, have, do*, the corresponding 3SG inflectional forms are respectively: *is, has, does*. On the other hand, Chinese has no tense/person agreement inflections. In this way, there is no verbal inflection to mark 3SG. For example, if sentence (2) is interpreted with a present simple tense, the verb *‘zhu*’ still appears with a bare form without any morphological inflections.

(2) John zhu zai Beijing.
*John live in Beijing.*
  ‘John lives in Beijing.’

#### Plural Marking in English

Nouns in English should be either singular or plural (Rullmann and You, 2006). According to Cazden (1968), there are two obligatory contexts for plural markings: linguistic and contextual hints. First, plural markers are compulsorily required after linguistic cues, which contain determinatives (i.e., *some*), quantified numbers (i.e., *two*), noun phrases (i.e., *a group of*), and plural demonstratives (i.e., *those*). Second, nouns can be specified with sentential discourse or pictorial contexts, which are also called as contextual cues. Nouns can be inflected for a plural marking in two ways: regular and irregular inflections. Affix ‘*-s*’ is used to mark regular plural nouns, while there are four main morphological inflections for irregular plural marking, namely, vowel change (i.e., *foot–feet*), no change (i.e., *Chinese–Chinese*), ‘*-en’* suffix (i.e., *child–children*), and suppletion (*mouse–mice*).

In addition, it is necessary to identify incorrect language uses when analyzing L2 learners’ acquisition of plurals (Cazden, 1968; Jia, 2003). In obligatory plural contexts, omission is the most common error type. Additionally, the overgeneralization of regular plural inflections also occurs by means of adding affix ‘*-s*’ to a irregular noun (i.e., childs*), to an inflected irregular noun (i.e., childrens*), or to a noun with singular and plural isomorphism (i.e., Chineses*). As for the non-obligatory plural contexts, morpheme ‘*-s’* may be suffixed to a singular noun, such as ‘an apples*’, or to an uncountable noun, like ‘waters*’. A further inappropriate inflection is that adding ‘*-s’* to an adjective or quantifier, for instance, ‘greens*’ or ‘alls*’.

#### Plural Marking in Chinese (‘*men*’ for Nouns With Human Property)

According to Corbett (2000) and Zhang (2008), there is no specific plural marking in Chinese, and thus Chinese bare nouns have general number, which can be considered as either singular or plural, for instance, ‘*xuesheng*’ in sentence (3) can be interpreted as *students* or *the student* depending on the context.

(3) Xuesheng zou le.
  student(s) leave LE.^2^
  ‘The student(s) have/has left.’

On the other hand, Chinese is a classifier language, which means a classifier is required when expressing the number of a noun. For example, sentence (4) would be ungrammatical if the classifier ‘*ge*’ is removed in Chinese.

(4) San ge xuesheng
*Three ge-Cl student.*
  ‘Three students’.

Although Chierchia (1998) argues that classifier and plural systems can not coexist in the same language, many classifier languages can obtain plural markings. For example, Korean and Persian are classifier languages that have plural markings. Bengali is also a classifier language with two plural morphemes: *-gulo* for a collective reading and *-ra* for an associative reading (Biswas, 2013). Also, ‘*men*’ in Chinese is regarded as a plural morpheme (Li, 1999). However, compared with ‘*-s*’ in English, there are some constraints for ‘*men*’ to mark plurals. First, ‘*men*’ must follow a noun with a human property. Second, ‘*men*’ is incompatible with numeral values. Third, the plural form with ‘*men*’ must be definite, and it is, therefore, ungrammatical in the existential construction like sentence (5). Last, all pronouns can be followed by ‘*men*’, and it is also different from the marking of English pronoun plurals, as example (6) showed. For example, plural ‘*you*’ is not morphologically inflected in English, while *‘ni’/‘you (single)’* should be followed by ‘*men*’ to present a plural feature in Chinese.

(5) you ren men*.
*There people men.*
  ‘There are people.’

(6) Wo men ni men ta men
*I men you men she/he men.*
  ‘We’ ‘you (Pl)’ ‘they’.

**Figure 1:**
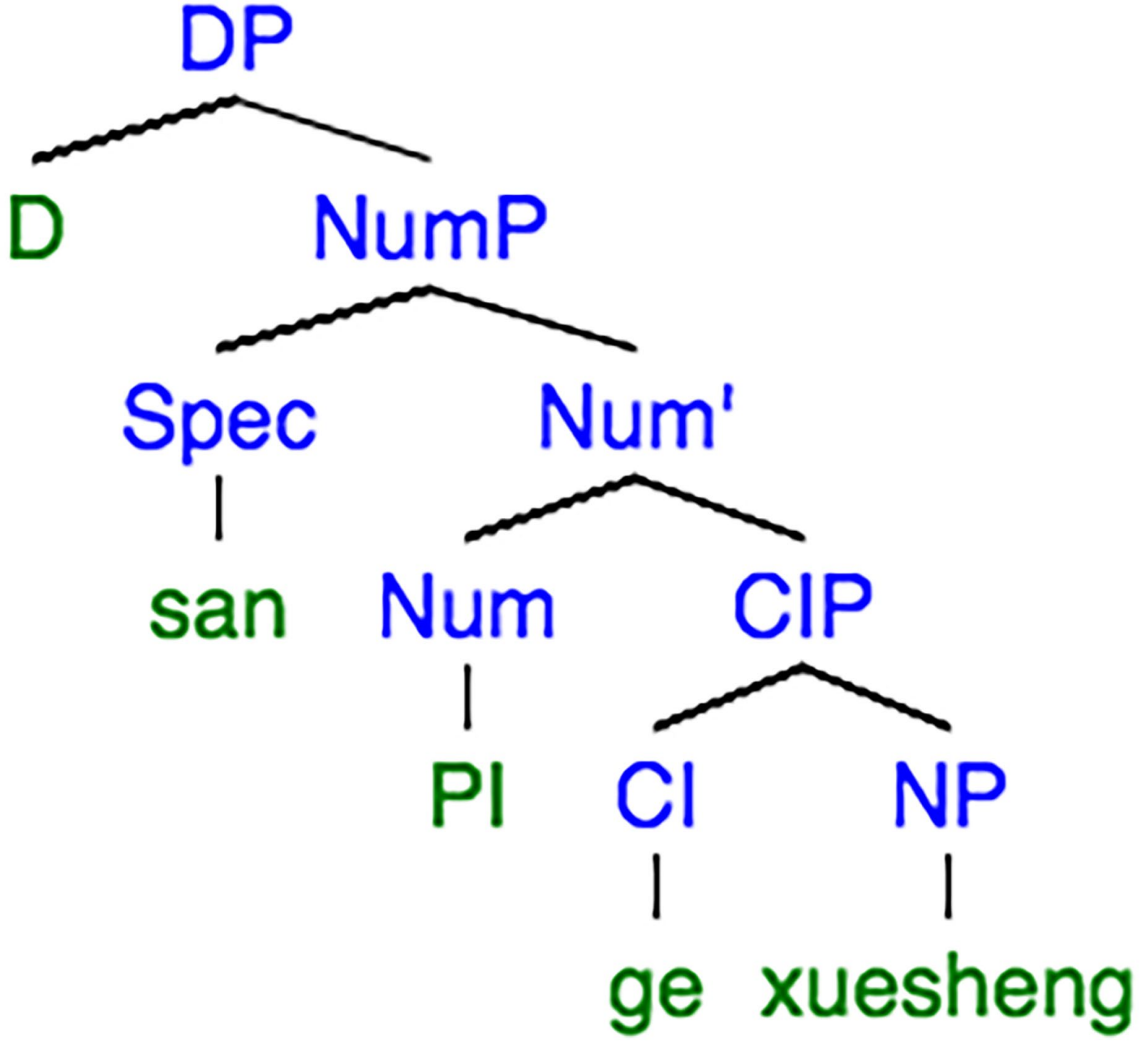
Syntactic tree extracted from Li (1999, p. 86).

**Figure 2:**
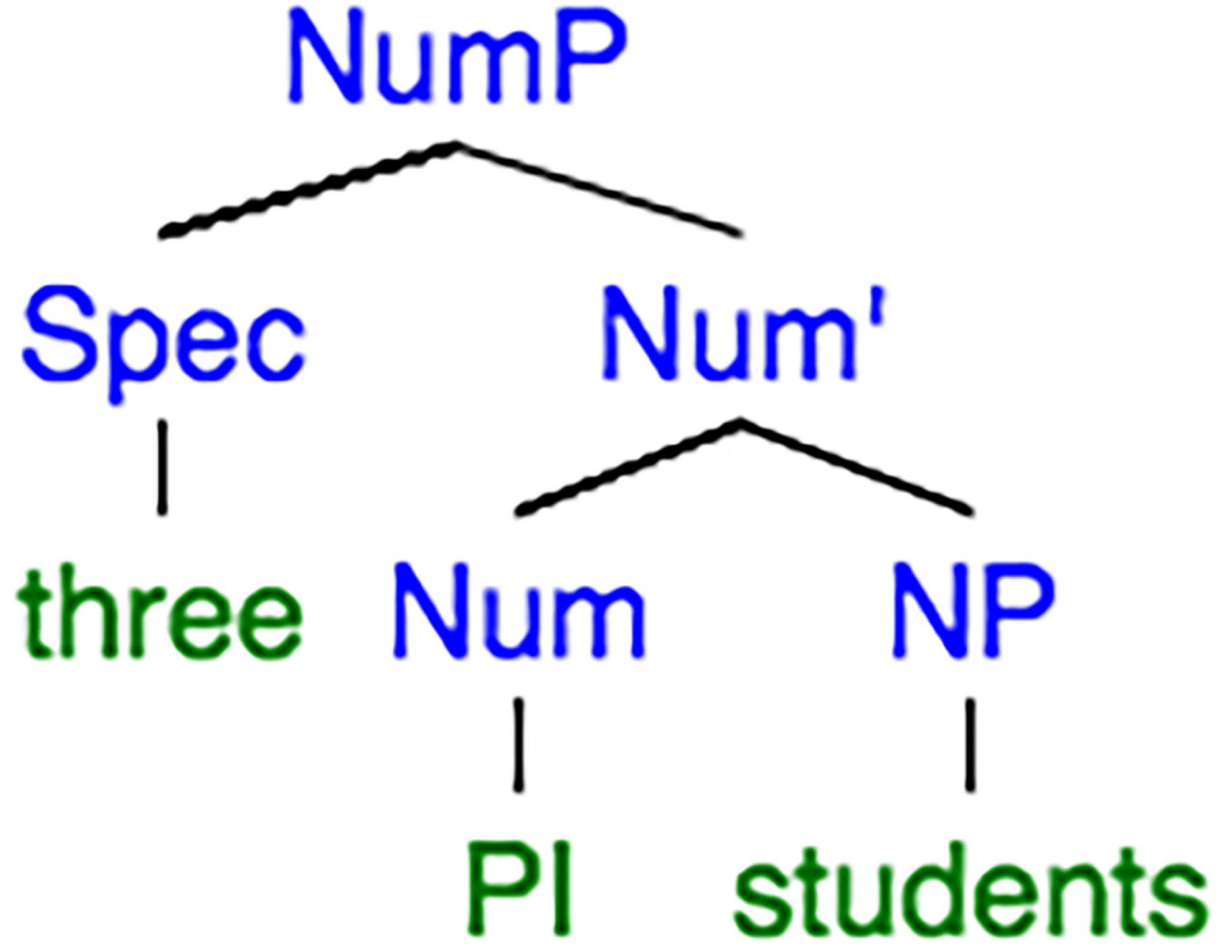
Syntactic tree extracted from Li (1999, p. 87).

As for a classifier language, the plural (Pl) feature should be interpreted in the item in Determiner (D), while a non-classifier language expresses plurality in the item at Noun (N) level. On the other hand, the plurality is initiated in Number (Num) node no matter in a non-classifier language or a classifier language. The differences and similarities of plurality between a non-classifier and a classifier language can be discerned from a clearer view of the syntax trees of ‘*three students*’ in Chinese (Figure 1) and English (Figure 2).

**Figure 3:**
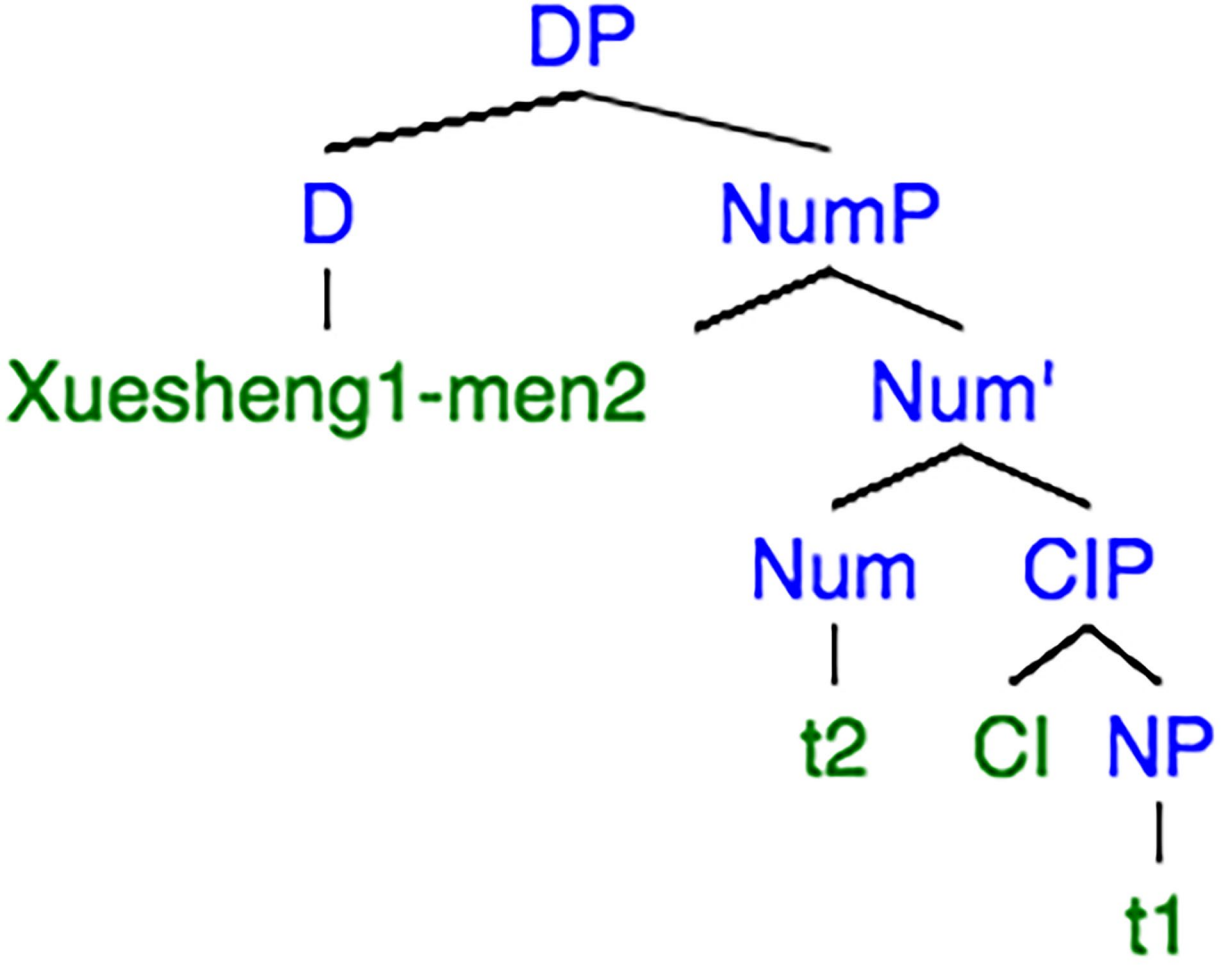
Syntactic tree extracted from Zhang (2008, p. 411).

In Chinese, when ‘*xuesheng’* compounds with ‘*men’*, the corresponding syntax tree is shown in Figure 3. In this case, ‘*men’* is placed next to a common noun-‘*xuesheng*’ in D to mark plurality, since the Pl feature in Figure 1 cannot directly move across Cl head to NP and the only option left for the plural feature is to be raised to D. When a noun is combined with *‘men’*, it must be definite. Hence, *‘xuesheng’* is raised from NP to D through an empty Cl and Num. According to the Head Movement Constraint (Chomsky, 1986), a head cannot be raised through another head. Thus, if N needs raised to D, the head in ClP and NumP must be absent. In this case, *‘-men’* is incompatible with the classifier and the numeral. In contrast, a counterpart in English can have plural inflections, such as ‘*glasses*’ in ‘*three glasses of water*’. However, this account should be treated with caution, since the ‘*glasses*’ in English is likely to be N category, while the classifier is an independent category in Chinese.

To sum up, ‘*men*’ in Chinese is not a simple counterpart of ‘-*s*’ in English, although both of them are suffixes which cannot be used alone. In addition, the use of ‘*men*’ is limited in Chinese; for instance, it can only be attached to the nouns with human nature and cannot coexist with the [number classifier] construction. Furthermore, the inflection ‘*men*’ is not mandatory even with human nouns, but ‘*-s*’ is compulsory for regular countable nouns in the plural context. However, it is worth mentioning that ‘*men*’ is an item which can be used to value the uninterpretable plural features on the functional heads of [Number] (Zhang, 2008). Consequently, as for the verbal inflections of 3SG, Chinese has no corresponding morphological system at all. But there potentially is a connection between syntax and morphology for nominal inflections due to the Chinese plural morpheme ‘*men*’, and this relationship is similar to plural affix ‘*-s*’ in English.

### Research Question and Prediction

As mentioned above, Chinese learners tend to omit affix ‘*-s*’ both in the contexts of 3SG and regular plural nouns. Most research attributes it to the L1 transfer involving with prosodic features, the cognitive process, or the missing surface morphology, while few included the discussion on the verbal syntactic property. As for the insufficient plural marking ‘*-s*’, it mainly referred to the influencing factors, such as the initial age of first exposure to L2, language environment, and mental processes. Enlightened by the relation between copula ‘*be*’ in English where Chinese learners have no inflectional difficulty and its counterpart ‘*shi*’ in Chinese, ‘*-men*’ is found to be more appropriate to test the L1 impact of morphological inflections, since ‘*shi*’ does not involve any inflectional forms whereas ‘*-men*’ can be attached to a human noun to constitute a plural marking like the plural affix ‘*-s*’ does in English.

In short, as regards verbal inflections, there is no agreement marking for 3SG in Chinese, while the morpheme ‘*-s*’ should be affixed to regular finite verbs for 3SG in English. However, as for nominal inflections, ‘*-men*’ functions similarly to ‘*-s*’ in English when it is combined with human objects. To figure out whether the L1 syntactic properties can have an influence on the acquisition of L2 tense and agreement morphology, the comparison between affix ‘*-s*’ for nouns and verbs was observed and analyzed in the spoken and written forms, separately. Meanwhile, the correlated hypothesis (the RDH) can be tested, as the following research question arises:

(1) Whether L1 Chinese learners of English will show higher suppliance rates for 3SG ‘*-s*’ than plural *‘-s*’ in both spoken and written discourses?

In terms of the RDH, if there is no corresponding morphosyntactic feature in the native language, L2 learners are not able to complete the computational process to produce morphology or phonology in the target language. With regard to the research question in the present study, if the RDH is correct, the Chinese participants will provide more *‘-s’* morpheme for regular plural inflections than for 3SG verbal markings, since Chinese has no person and number agreement verbal inflections with 3SG context, while it has the plural morpheme *‘men’* to mark human nouns or pronouns in some cases.

## Methodology

### Participants

Thirty-six Chinese postgraduate students studying at a public university in the UK were recruited as participants in this empirical research. Three of the students participated in the pilot study, and the rest of them undertook the main experiment. To adhere to the ethical rule of privacy, all of the participants’ names have been anonymized by numbering them in the order they undertook the tasks. To distinguish the participants in the main study from the pilot study, the former was labeled with ‘No. + Number’ (i.e., No. 01) while the latter was marked with ‘P + Number’ (i.e., P01). To reduce the possibility of pre-knowing what would be tested in the research, none of the participants had taken the second language acquisition module. Hence, they studied in various departments except for the School of English. As for L2 proficiency level, all participants obtained an overall IELTS score ranging between 5.5 and 6.5, which corresponds to B2 level according to CEFR (Common European Framework of Reference). No necessary band for each sub-skill score was required, even though the information was collected in the personal detail sheet (See Appendix 1). There were nine students with an overall score of 5.5, nineteen with 6.0, and eight with 6.5. The average length of time in learning L2 English is 14 years with a standard deviation (SD) of 1.4. Nine participants lived in an English-speaking country for more than 1 year. The remaining twenty-seven students had lived in the UK for more than half a year but less than 1 year.

### Experimental Tasks

It is necessary to analyze learners’ use of morphology from more than one task type (Bardovi-Harlig, 1995). Harklau (2002) reports that it is crucial to collect both spoken and written data to investigate the acquisition process. On the one hand, participants may not react consistently under different task styles. To determine whether style-shifting would have an influence on students’ performance on L2 morphological and syntactic properties, Tarone (1985) tested the use of four linguistic features: 3SG, plural *‘-s’*, articles, and direct object pronouns under three different tasks: a written grammar test, an oral interview, and an oral narrative task. It was observed that except for plurals, subjects performed variably in the use of other three items in different tasks. Hence, Tarone (1985) concluded that L2 learners presented different performance on different tasks for different tested items. Ellis (1987) then reached a similar conclusion by researching past tense inflections of regular, irregular, and copula verbs with three different tasks (planned writing, planned speech, and unplanned speech). The results found that the subjects’ use of irregular verbs was little affected by task styles, whereas participants marked regular past tense differently among all three tasks, and copulas were inflected differently between the two speech tasks.

Due to the phenomenon presented in the above research, it is understandable to regard the investigation style as a factor which may have an effect on L2 learners’ performance. On the other hand, a written task can compensate for the difficulty in pronunciation in a spoken task (Wolfram, 1989). This means that if research only focuses on the data from a spoken task, it may lead to a limited access to learners’ interlanguage grammars, or vice versa when students are able to pronounce the sounds but lack the knowledge of the corresponding orthographic representation. Hence, to increase the validity and generality of the results, there are two task types with spoken and written discourse in the current study.

#### Semi-constructed Conversation Task

Aligned with previous research, a conversation between the researchers and the participant was conducted to elicit affix *‘-s’* in L2 learners’ oral production (Dulay and Burt, 1973; Jia, 2003; Hsieh, 2009; Blom et al., 2012; Sari et al., 2015; Lardiere, 2017). In order to effectively utilize the time during the conversation, the researchers prepared questions (Appendix 2) in advance. In this way, the interlocutors did not need to spend much time thinking about what they should talk about, as the questions were ready there. In order to collect the inflections for 3SG and plural *‘-s’* as much as possible, most questions were presented in simple present tense, for instance, daily routines and hobbies of a person. There were 23 questions in total with five topics, namely, your mother, a best friend, a favorite teacher, a favorite celebrity, and a pet. As can be seen, these topics were closely related to people’s daily life so that the participants were able to feel comfortable and free to express their opinions when communicating with the researchers. The overall duration for the conversation was expected to be about 5–10 min. Furthermore, some communication strategies were applied to deal with incidental circumstances. For example, if it had not taken 5 min after all the questions were answered, the researchers would add more questions, such as ‘do you know what Mariah usually does?’ in No. 06 (Mariah is a friend for the researchers and No. 06). However, the researchers would not interrupt the speech or reduce the number of questions if the conversation had exceeded 10 min. The longest time among the thirty-six spoken tasks (3 pilot and 33 main experiments) was 8 m 15 s. Besides, every participant responded to all five topics within 10 min. As for the topic of ‘a pet’, considering that some participants may not own a pet or they have more than one pet, the researchers would skip to the next topic or ask them to choose one of pets to describe. Hence, not every conversation would cover all 23 questions.

#### L1-L2 Translation Task

In this study, a translation task from Chinese to English was presented as a written task. A majority of research only focused on spoken data to analyze subjects’ performance in making morphological inflection of affix *‘-s’* (Dulay and Burt, 1973; Goad et al., 2003; Jia, 2003; Blom and Duncan, 2012), while little research has been collected on the written data. Ionin and Wexler (2002) conducted a grammatical judgment test to support the missing surface inflection hypothesis in terms of tense and agreement morphology. In Sari et al. (2015) study, 40 Turkish subjects were required to write daily routines of their family as homework. According to House and Blum-Kulka (1986), a translation task can lead to plentiful explorations, since it involves processing two languages and cultures in terms of linguistic, textual, and social systems. The translation task was adopted with two advantages. First, it saved time, as the structure and content were already provided in native languages. Second, it could be regarded as a supplementary method when students had not enough opinions to talk about (Frerch and Kasper, 1986). Hence, referring to Sari et al. (2015) research, the written task was adapted to be a translation task with the topic of ‘my mother’s daily routine’ in the present study (see Appendix 3), and the corresponding transcription can be seen in Appendix 4. There were about 190 words in the translation task. To ensure that the subjects were aware of the context of a simple present tense, an explanation about daily routine was added in the instruction part. In addition to the instruction, there was a frequency adverb ‘*tong chang’/‘usually’*, which is commonly used in a simple present tense in the first sentence. Frerch and Kasper (1986) point out that it is possible for participants to replicate the model or structure of the sample sentence provided in the instructions, which means that the translated sentences are likely to be produced as an imitation of the sample sentence rather than their own minds. Therefore, there was no sample sentence in the written task. Moreover, in order to prevent the participants from getting target inflections in the dictionary, no type of dictionaries was allowed in the whole translation task. To reduce the complexity and difficulty of the original text, word and sentence structure was designed to be as easy as possible to be understood. The subjects would be given enough time for the translation to reduce the pressure from the time limitation (Ellis, 2005). To trace any self-correction, a pen which could not be erased was provided for each participant.

### Pilot Study

Before the main study is carried out, it is imperative to run a pilot study to diagnose whether the provisional methods are appropriate and to foresee any variables that may affect the validity of the results (Van Teijlingen and Hundley, 2001). The role of a pilot study is to reduce the risk of potential practical problems rather than to get rid of all the confounders, as there are many other factors that cannot be easily tested or controlled in the real test (MacIntyre and Gardner, 1994; Dörnyei and Schmidt, 2001). Bedsides, the data of the pilot study should be excluded when doing the analysis for testing the hypotheses (Peat et al., 2002) state that. In other words, the researchers only used the data of the pilot study to decide the effectiveness and appropriateness of the research projects. Three participants who got an IELTS overall score of 5.5, 6.0, and 6.5, respectively, were invited to do the pilot studies.

As for the spoken task, the pilot study exerted a great influence on modifying the formal experiment design as there were two different task versions before the final conversation task was decided. A picture description task with the topic of ‘a man’s daily life’ was chosen at first as other researchers did (Goad et al., 2003; Jia, 2003). However, in the first pilot study, the results showed that all three participants failed to produce enough morphological inflections for 3SG, even though there was a predetermined context for the target tense in the instruction. Instead, they tended to use the structure of ‘*will do*’ with the future tense. The general results of the spoken task in the first pilot study shows that the subjects used the future tense more than the present tense, which only appeared one or two times. Turning to the number of plurals, except for P01, both P02 and P03 failed to provide sufficient countable nouns for marking plurals. Consequently, the researchers changed the picture description to a natural speech with the topic of ‘describe an intelligent people’ as the oral task. However, it was also rejected, as the participants were likely to describe the prior experience that they shared with the person they referred to. In this way, the oral production was largely occupied with the past tense. It indicates that there were few contexts for 3SG inflections, even though it was moderately higher than the data from the previous picture description task. In regard to the number of plurals, except for P02, two other participants made enough countable nouns. Due to the insufficient number of contexts for 3SG, topic description task was abandoned, and the researchers adopted the conversation task to collect oral data, as Blom et al. (2012), Hsieh (2009), and Jia (2003) did. Those researchers chose a conversation with a series of things happening in daily life, for instance, friends or schools, which was discussed above in “Semi-constructed Conversation Task.” All three participants provided enough contexts for marking the affix ‘*-s*’ of both verbs and countable nouns in this pilot study. Therefore, the semi-controlled conversion was identified as the spoken task in the main experiment.

As for the translation task, it got a satisfactory result in the first pilot study. A certain number of contexts required for inflecting 3SG and plurals were produced as expected, and thus it has been chosen as the task for written data.

As for the sequence of the two tasks in the present study, there was an accidental case which helped the researchers to decide the oral task should be conducted before the written task. The participant (appeared neither in the pilot study nor the main study) completed the written task first, and then he copied the sentences from the translation task to answer the question in the conversation. For example, when he had translated the question *‘what does your mother usually do on weekdays?’* he answered several sentences which were similar to the sentences he encountered in the written task. As a result, the spoken task was carried out before the written task to avoid participants from being negatively affected by repeating the translation text.

### Procedure

Participants completed the tasks individually in a seminar room. The participant first read the information sheet (Appendix 5), which included the main aim of the research, the requirements for the participants, and the general process of the two tasks. A personal detail sheet was presented then (Appendix 1), where the participants were required to provide their basic demographic information. The linguistic experience as second language learners was also contained, for instance, the length of learning English, their latest IELTS scores, and other information related to any second language learning. Second language achievement is partially correlated with the degree of anxiety. The correlation may be negative or positive, pervasive or subtle (MacIntyre and Gardner, 1994). Therefore, there was a quick chat between the researchers and the participant to reduce nerves before the spoken task Also, there was a short break between the spoken and the written task where subjects had an opportunity to ease the burden on their cognitive process before the subsequent translation task. The oral tasks were recorded by a sound-recording device with the permission of each participant.

### Data Analysis

The data from the conversations was first transcribed sentence by sentence and then double-checked as a whole document. The amount of the compulsory contexts of adding the suffix *‘-s’* for 3SG thematic verbs and countable nouns, and of inflecting auxiliary verbs (*have, do* and *be* verbs) with and without pronouns were counted separately. In order to have a more comprehensive understanding of the learners’ competence in adding *‘-s*’ to mark verbs with 3SG subjects, the researchers put auxiliary verbs into consideration, as suppletion and stem alternation are also a way to inflect verbs of 3SG. In addition, the combination of pronouns and inflected auxiliary verbs were also recorded as it can be regarded as a chunk in English (Kim and Ann, 2008), which bears an ‘empty’ syntactic lexicon (Lenci et al., 2001). As for the written task where the content has been set in advance, there were only three categories required be counted: thematic verbs, auxiliary verbs (i.e., *have, do, and be*), and plural nouns. The suppliance number of each inflection was calculated, and the raw frequencies were converted into percentages, which was an easier way to understand (Brown and Rodgers, 2002). All the results were analyzed on the statistical software SPSS (Statistical Package for the Social Sciences).

## Results

To eliminate the potential effect of L2 proficiency level on supplying suffix ‘*-s*’ for 3SG verbal inflections and plural nominal infections, the corresponding operations on SPSS were carried out. Taking the verbal affix ‘*-s*’ in the spoken data as an example, a One-simple K-S test was conducted first to identify that the suppliance rate for each group conformed to the normal distribution (Asymp. Sig > 0.05), and then a one-way ANOVA test showed that there was no significant difference not only among the three groups with different overall IELTS scores, but also for pairwise comparisons (Sig > 0.05). The same statistical operations were then repeated three times for plural markings ‘*-s*’ in the spoken task, and for 3SG and plural inflection ‘*-s*’ in the written task, respectively. The results indicate that the different IELTS grades ranging between 5.5 and 6.0 are not an influencing factor for the morphological inflection performance on supplying 3SG ‘*-s*’ and plural ‘*-s*’ in either the spoken or written task. As a result, there is no need to group the students with different IELTS scores when analyzing the data.

### Semi-constructed Conversation

#### General Result

Table 1 below illustrates an overall performance on supplying verbal inflections with a 3SG feature and nominal inflections with a plural feature in the conversation task. As for the suppliance rate, the lowest is the thematic verb inflections for 3SG contexts with 29.4%, and the highest is irregular plural markings with 100% (there was only one case for irregular plural inflection: ‘*children*’ in No. 06). Similar to the irregular plural, 99.4% ‘*be*’ verbs with 3SG (demonstrative or personal) pronouns are highly inflected in the obligatory context, which is followed by 3SG copula ‘*be*’ without pronouns with 93.2%. The fourth highest suppliance rate belongs to plural ‘*-s*’ markings as a chunk, which is 90%, and that is a little higher than the auxiliary verb ‘*have*’ with 85%. It should be noted that the total amount of regular plural inflections as a chunk is ten, which is relatively deficient. In addition, ‘*-s*’ for plural markings is the second lowest inflected rate with 75.9%. As can be seen, the biggest problem that the participants faced is supplying the affix ‘*-s*’ either for 3SG verbs or plural nouns, which accounted for the two lowest suppliance rates. Moreover, the difference between the affix ‘*-s*’ suppliance in marking 3SG verbs (29.4%) and plural nouns (75.9%) is significant as determined by paired-samples *t*-test (Sig. = 0.000 < 0.05). To calculate the effect size in paired-samples *t*-test, Cohen (1988) invents a formula named Eta Squared.^3^ The result demonstrates that the significant difference between 3SG *‘-s*’ and plural ‘*-s*’ is quite large (*η*^2^ = 0.68 > 0.14). In the next section, more detailed information about the students’ performance on inflecting words in 3SG and plurals for the oral task will be reported.

**Table 1:** Suppliance in all 3SG and plural markings in the spoken task.

Context	Third person singular				Plural		
Word type	Thematic verb	Aux. V.^*^ ‘*have*’	Copula ‘*be*’ without pronouns	Copula ‘*be*’ with pronouns	Regular plurals	Regular plural nouns as a chunk	Irregular plurals
Suppliance number	78/265	34/40	126/133	311/313	305/402	9/10	1/1
Suppliance rate	29.4%	85%	93.2%	99.4%	75.9%	90%	100%

* Auxiliary verb (Aux. V).

#### Third Person Singular Verbal Marking

In regard to the 3SG obligatory context, thematic verbs marked by adding the suffix ‘*-s*’ was supplied the least. Hence, it demonstrates that the participants had more difficulty in supplying affixation than suppletive inflections, and this phenomenon is consistent with the data in the prior studies (Lardiere, 1998a,b; Goad et al., 2003; Hawkins and Liszka, 2003; Goad and White, 2006; Lardiere, 2017). As for the auxiliary verb inflections, the participants perform better in supplying copula ‘*be*’ (99.5% with pronouns and 93.2% without pronouns) than auxiliary verb ‘*have*’ (85%). Moreover, there is a significant difference in marking 3SG copula ‘*be’* verb collocating with pronouns (99.5%) and without pronouns (93.2%; Sig. =0.046 < 0.05 in a Wilcoxon signed-rank test).^4^

In addition to the omission of 3SG markings, the participants overused 3SG verbal inflections in non-3SG contexts, as shown in Table 2. The overgeneralization of affixal 3SG markings was found seven times in total, which includes three times with the first person pronoun, such as I likes*… (No. 03), two times with a plural noun subject, like the teachers tends* to … (No. 29), one time with the third person plural pronoun in the example of they makes* … (No. 06), and two for an infinitive verb ‘to updates*’ in No. 12. Similar with thematic verbs, auxiliary ‘*be*’ verb were also commonly overgeneralized, which appeared seven times. The participants tended to put an unnecessary ‘*is*’ between a 3SG subject and a bare thematic verb in a positive sentence. For example, the sentence structure, like ‘she is* very love me’ (No. 18), occurred seven times. Additionally, the students wrongly provided the 3SG copula ‘*is*’ for a plural subject, such as ‘my parents is* doctors’ (No. 22).

**Table 2:** Incorrect uses of 3SG verbal inflections in the spoken task.

Verbal types	Context	Example	Frequency
Thematic verbs Thematic verbs	First person pronoun	I likes*…	3
	Third person plural pronoun	they makes* …	1
	Plural noun	the teachers tends* to …	1
	Infinitive form	to updates*	2
Copula ‘*be*’	S-V-O^*^ in positive sentences	she is* very love me	7
	Plural noun	my parents is* doctors	5

* S-V-O represents a sentence structure of Subject–Verb–Object.

#### Plural Marking

As can be seen in Table 1, the affixation ‘*-s*’ (75.9% not in a chunk and 90% in a chunk) was supplied less than irregular plural markings (100%), However, as the irregular plural marker appeared only once, this statement should be treated with caution. As for the regular plural markings, the participants produced some plural nouns in a chunk, such as *‘watch movies’* (No.30). Only one of ten (10%) plural markings in a chunk was not inflected, that is ‘make note*’ in No. 10. By contrast, 24.1% regular plural markings are omitted in a non-chunk expression.

In terms of the incorrect suppliance of plural ‘*-s*’, there were six types as shown in Table 3. The type of adding a ‘*-s*’ to a singular noun constituted the majority, which accounted for two-thirds of all incorrect plural uses. In addition to nouns, the plural affix ‘*-s*’ was overgeneralized to adjectives and reflexive pronouns. Among them, the structure as ‘a four years* old woman’ (No. 06) was produced three times. The lack of plural marking knowledge may not the main reason for the subjects’ failure to provide the grammatical structure. Instead, the students might misuse plural markings because there was a numeral indicator ‘*four*’ before the noun. In this way, it was assumed that the participants were not aware of the adjective structure for expressing age, where a bare noun ‘*year*’ should be linked with ‘*four*’ to serve as a modifier. However, more research are required to determine whether the absence of the adjective structure knowledge is the cause. As for nouns in a chunk, only one participant (No.02) made a redundant ‘*-s*’ in the case of ‘*for examples*’.

**Table 3:** Incorrect uses of plural ‘*-s*’ in the spoken task.

Context	Example	Frequency
Singular	One students*	21
Uncountable nouns	knowledges*	4
Irregular plural nouns	Childrens*	3
Adjective	A four years* old woman	3
Reflexive pronouns	Himselfs*	1
collocations	For examples*	1

### Translation Task

#### General Result

The data for the written task was not analyzed as a contrast to the spoken task, as this study does not aim to compare the performance between the different task types. Based on the content of the original text, four types of verbal and nominal inflections are counted in total, namely, 3SG inflections for thematic verbs and auxiliary verbs, and plural markings with regular and irregular inflections. It can be seen in Table 4 that the participants had a near native-like proficiency in marking 3SG auxiliary verbs with no omission. The second highest suppliance rate in the written task was the irregular plural marking: *teeth* with 92%, which was a little higher than regular plural affixation with 91.35%. 3SG thematic verbs were inflected at least with the rate of 74.1%. As for the different performance of 3SG ‘*-s*’ and plural ‘*-s*’, Wilcoxon signed-rank *t*-test demonstrated that there was a statistical difference between them with Sig. =0.004 < 0.05.

**Table 4:** Suppliance in all 3SG and plural markings in the written translation task.

Context	Third person singular		Plural	
Word type	Thematic verb	Aux. V	Regular plural	Irregular plural (*teeth*)
Suppliance number	347/468	109/109	334/347	23/25
Suppliance rate	74.1%	100%	91.35%	92%

#### Third Person Singular Verbal Marking

The 3SG marking for auxiliary verbs was fairly high in the written task where all the participants were able to give correct inflections for all auxiliary verbs. Hence, the irregular inflections achieved a higher marking rate than the regular inflections for thematic verbs where 25.6% of 3SG ‘*-s*’ were omitted. Moreover, as shown in Table 5, the overgeneralization of 3SG ‘*-s*’ was found four times, and three of them appeared in the form of ‘I goes*’ (No. 6, 13, 15) with the first person pronoun. Another one was produced with a plural subject, which was ‘she and her friends goes* shopping’ in No. 16. Although all the participants can correctly inflect all auxiliary verbs with the 3SG feature, No. 17 wrote a sentence: *if it is rains*, where ‘*is*’ should have been deleted. However, this case can also be regarded as an incorrect use of the thematic verb ‘*rains*’, since if ‘*rains*’ was replaced with the adjective ‘*rainy*’, the sentence would be grammatically correct.

**Table 5:** Incorrect uses of 3SG verbal inflections in the written task.

Verbal types	Context	Example	Frequency
Thematic verbs	First person pronoun	I goes*…	3
	Plural noun	She and her friends goes* shopping	1
Copula ‘be’	S-V-O in positive sentences	It it is* rains	1

#### Plural Marking

In regard to the plural marking rate, the irregular plural (92%) was slightly higher than regular plurals (91.35%) as Table 4 shows. Also, this comparison needs to be taken with caution as in the spoken task. First, the huge disparity of the sample number between the irregular markings (25) and regular markings (347) may contribute to the statistical unreliability. Second, there was only one irregular noun ‘*tooth–teeth*’ required to be inflected in the whole task. Hence, it seems meaningless to contrast the suppliance rate between these two kinds of plural markings. Concerning the irregular noun *‘tooth–teeth’*, eight participants did not translate the verbal phrase ‘*shua ya’/‘brush teeth*’ at all. The majority of the remaining twenty-five students provided the correct plural form ‘*teeth*’, while two of them failed to do so. No. 08 and No.02 wrote the bare noun ‘*tooth*’ without any morphological inflections. It is worth mentioning that the low proficiency level in No. 02 might be the cause for the omission of the irregular plural markings. From the translation data of No.02, even simple words ‘*two*’ and ‘*always*’ were misspelled as ‘tow*’ and ‘alway*’. As for regular plural markings, the marking rate (91.35%) was based on a relatively large sample of 347, and there were a variety of nouns needed to be suffixed with ‘*-s*’.^5^

With regard to the incorrect uses of the plural morpheme ‘*-s*’, there were three error types occurring in the written data, as presented in Table 6. Five incorrect uses occurred in uncountable nouns, ‘foods*’ accounted for four and ‘milks*’ (No. 27) for one. No. 21 wrote ‘dogs*’, whereas there was only one dog referred in the original text. Moreover, No. 10 marked the plural affix ‘*-s’* to the irregular noun ‘*tooth*’, which was shown as ‘toothes*’. In this case, it seemed that No.10 realized that ‘*tooth*’ should be marked with the plural feature, but overgeneralized the regular form to an irregular word.

**Table 6:** Overgeneralization of plural nominal inflections in the written task.

Context	Example	Frequency
Singular	dogs* (only one dog referred)	1
Uncountable nouns	foods*	5
Irregular plural nouns	toothes*	1

## Discussion

### The Comparison Between 3SG ‘*-s*’ and Plural ‘*-s*’

Based on the data shown in “Results,” the research question: whether L1 Chinese learners of English will show a higher suppliance rate for 3SG ‘*-s*’ than plural ‘*-s*’ in both spoken and written discourses, can be answered. There was a significant difference between the two morphological inflections with Sig = 0.000 (<0.05) in the spoken task and with Sig = 0.004 (<0.05) in the written task. In other words, the participants marked ‘*-s*’ for 3SG verbs considerably less than regular countable nouns regardless of the task type. Therefore, this result matches the prediction from the RDH, which indicates that the absence of the functional feature in L1 will impoverish the acquisition of the corresponding feature in L2. L1 Chinese lacks agreement verbal markings, while it adds the morpheme ‘*men*’ to a human noun or a pronoun to mark a plural feature. In terms of the computational procedures in language faculty, it means that Chinese participants have difficulty in encoding the uninterpretable [third person, singular] syntactic feature through inflectional morphology, while the acquisition of [plural], being an interpretable feature for nouns, can be facilitated by adding the morpheme ‘*-men*’, although this process is restricted to part of nouns in Chinese. Therefore, the process of presenting the [plural] feature in English nouns can be easily acquired, as there is a similar morphological inflection: affixation for *‘men*’ in L1, while this kind of facilitation for the [third person, singular] feature is absent. Hence, Chinese students will show more optionality in supplying 3SG thematic verb inflections than plural affixation in English. This conforms to the results of the present study where 3SG ‘*-s*’ were supplied less than plural ‘*-s*’.

### Initial L2 Exposure Age

In addition to the linguistic perspective, the RDH suggests that when the features are absent in native language, the age of exposure to L2, as a non-linguistic factor, also plays a critical role in determining the accessibility of morphosyntactic features. If L2 learners are not exposed to the target language at an early age, they will have permanent difficulty in presenting L2 morphological form, which is caused by the lack of the relevant interpretable syntactic knowledge in L1. The average age to start learning English was 13.94 years old with SD = 1.435. Hence, it is reasonable to infer that although the adult participants learned English for a certain period of time, the first exposure age was too late to attain the 3SG morphological inflections in their interlanguage.

### More Inflections for 3SG ‘*-s*’ for Individual Cases in the Written Data

**Figure 4:**
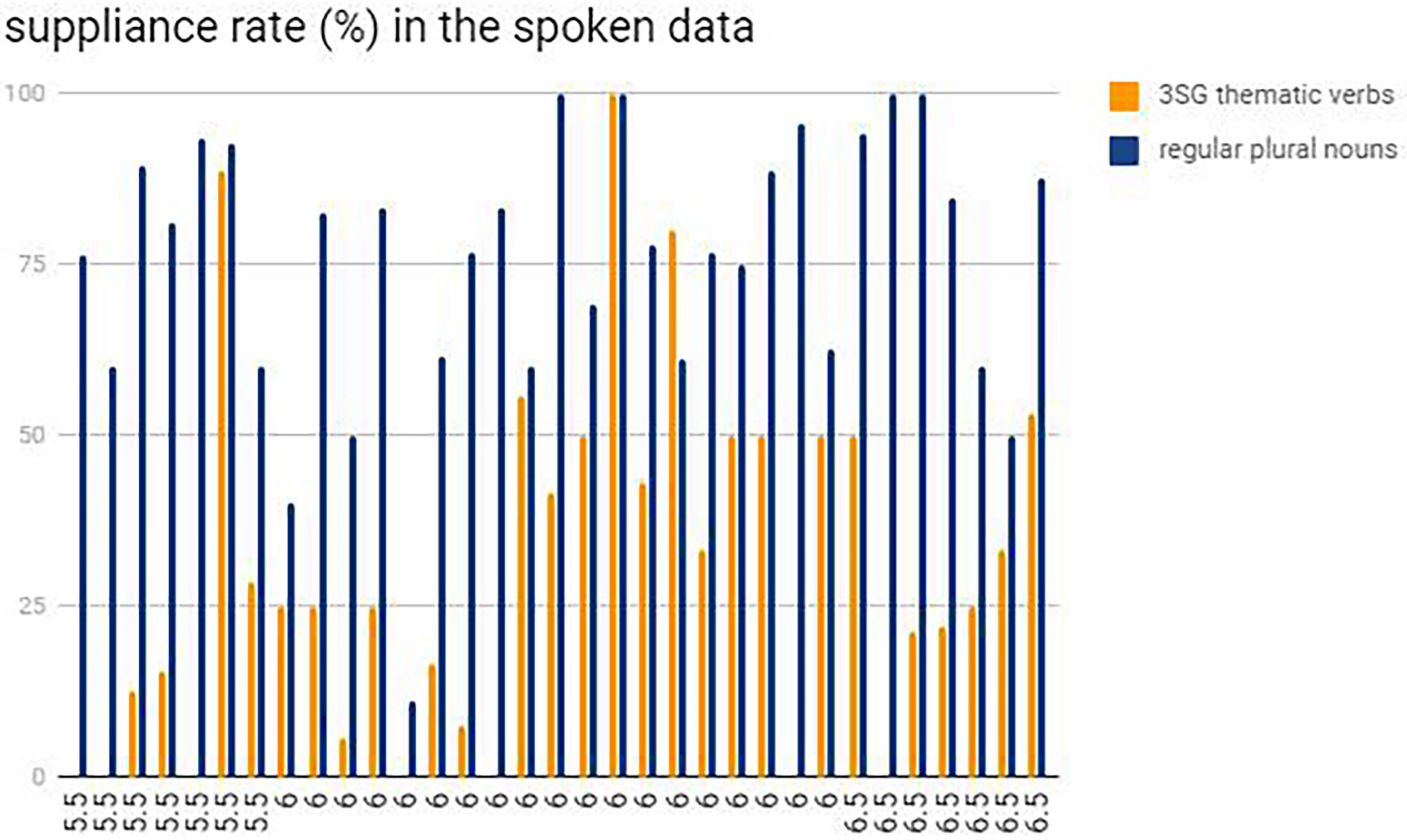
The comparison of suppliance rates between 3SG ‘*-s*’ and plural ‘*-s*’: individual results in the spoken data.

**Figure 5:**
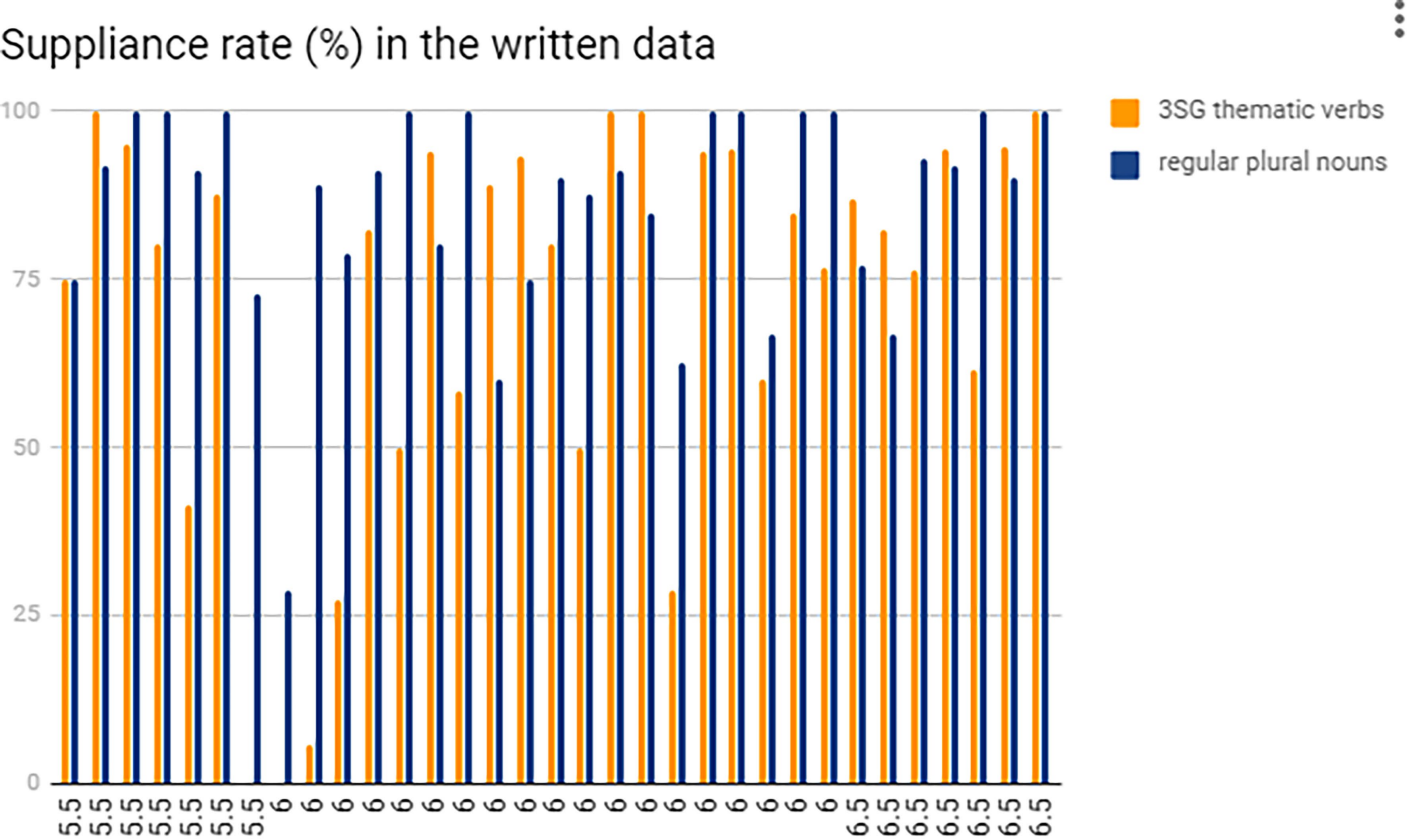
The comparison of suppliance rates between 3SG ‘*-s*’ and plural ‘*-s*’: individual results in the written data.

One interesting phenomenon was discovered when focusing on the differences between the individual performance of the plural and 3SG affixation markings in the two tasks. A great difference in marking ‘*-s*’ for nouns and verbs was observed in both the tests. However, all the participants had a lower suppliance rate in marking 3SG ‘*-s*’ in the spoken data, while ten participants marked less plural morphemes ‘*-s*’ in the written data. More intuitive information is revealed below in Figures 4, 5. As for the ten students who performed better in 3SG verbal affixations than plural regular marking in the written task, a half of them were distributed to the participants who got IELTS score of 6.0, and four who scored 6.5, while the remaining one got 5.5. It seemed that the participants who got higher IELTS scores had a tendency to mark more 3SG affixations, although the proficiency level between 5.5 and 6.5 for IELTS scores have little effect on each inflectional morphology (plurals and 3SG thematic verbs) as presented at the beginning of “Results.” Therefore, it seems contradictory to the RDH to some extent that these ten students marked more 3SG ‘*-s*’ than plural ‘*-s*’.

Nonetheless, again, this situation only occurred in the written task. Hawkins and Liszka (2003) stated that the different task modalities may cause different results. The output checking system was mentioned to explain why the written data always performed better than the spontaneous speech data for the past tense marking. Consequently, even though there were ten participants who marked affix ‘*-s*’ for 3SG thematic verbs than plurals in the written task, it may be the result of the output detecting process. Evidence can be found in the results from No. 03, where the plural feature of ‘*TV programs*’ was marked in the written task, but it was absent in the spoken data, as ‘TV program*’. Further evidence can be found in verbal inflections. For example, No. 29 still failed to inflect the auxiliary verb for 3SG as ‘she have* been …’ after having provided the correct form as ‘*she has been …*’ for three times in the spoken task. It may imply that the former three correct forms were simply resulted from the output checking system, since when the student was distracted from the checking process, they were likely to forget to supply the inflectional morphology. On the other hand, the RDH can give explanation to the individual cases to some extent. Due to the lack of the syntactic feature in the interlanguage grammar, the participants needed to rely on the output checking system to supply 3SG inflections. However, the output checking system does not work all the time. A pressure from a spontaneous conversation may increase the burden on the output checking system in the oral task. In this way, the phenomenon that 3SG ‘*-s*’ were inflected more than plural ‘*-s*’ only existed in the written data, since the participants were able to reply on a less-affected detecting system to mark 3SG features in the written discourse.

### More Overgeneralization for the Plural Affix ‘*-s*’

In addition to the omission of inflectional morphology, it is noteworthy that the overgeneralization of the regular 3SG and plural markings frequently appeared in copula verbs, and singular and mass nouns. In Table 7, we can see that the overgeneralization of ‘*be*’ verb is most common among all the incorrect uses of 3SG inflections. As regards the overgeneralization of 3SG copula ‘*be*’, Lardiere (2017) interprets that the L2 learners tend to associate the morphological finite feature directly to its functional category. In this case, the students do not select the unraised thematic verbs to inflect, and thus the raised ‘*be*’ verbs are chosen to bear the inflectional morphology. When it comes to the overgeneralization of affixal plural markings, Jia et al. (2002) suggest that it is more extensive in singular and mass noun contexts, which is consistent with the current experiment. In the spoken data (Table 3), almost two-thirds (21/33) of plural ‘*-s*’ overgeneralization appeared in a singular context. As for the written data (Table 6), five of the seven were incorrectly applied for uncountable mass nouns, which consisted of ‘foods*’ and ‘milks*’ for four times and one time, respectively.

**Table 7:** Overgeneralization of 3SG markings for the two tasks.

Verbal types	Thematic verbs				Copula ‘*be’*
Contexts	First person pronoun	Third person plural pronoun	Plural noun	Infinitive form	S-V-O In positive sentences
Frequency	6	1	2	2	7

As can be seen in Table 8, compared with 3SG ‘*-s*’, the overgeneralization for plural markings was more universal both in the spoken and written data. This comparison may also give supportive evidence for the RDH. Frist, the overgeneralized errors should not be learned from the language input (Tomasello and Herron, 1988). For example, it is rare for L2 learners to come across ‘foods*’ in their L2 input. Hence, the way the students produce the expressions, like ‘foods*’, is likely to derive from their own knowledge. Compagnon (1984) indicates that different knowledge from different native languages has an impact on the overgeneralization suppliance. All the participants’ native language is Chinese, where syntactic features about morphology is different for verbs and nouns. The RDH predicts that the plural morpheme ‘*-men*’ in Chinese can facilitate the acquisition of plural affix ‘*-s*’ in English, while this kind of facilitation is absent in 3SG verbal inflections due to the lack of agreement verb markings in L1. However, adding the suffix *‘-s’* is not the only way to mark the plural feature in L2. Accordingly, the overgeneralization of plural affixation markings to nouns should be found more frequently than the overgeneralization of regular 3SG markings in L1-Chinese subjects, since compared with 3SG ‘*-s*’, affixation for plural nouns is presumably much more remarkable in students’ minds.

**Table 8:** The comparison of the frequency of overgeneralization of 3SG ‘*-s*’ and plural ‘*-s*’ in the two tasks.

	Spoken task	Written task
3SG ‘*-s*’	15	1
Plural ‘*-s’*	33	7

In addition, there is one speculation about the reason for the overgeneralization of plural ‘-*s*’. The learners frequently overgeneralized the plural ‘-*s*’ marking to singular and mass noun contexts, which are contexts that are incompatible with ‘-men’ but are compatible with a classifier in Chinese. In this case, Chinese learners perhaps use ‘-*s*’ to mark plural nouns in addition to some other features or feature combinations in the noun phrase because of the negative transfer from the L1 plural system.

### From the Aspect of the Prosodic Pattern

The results in the present study show negative evidence for the PTH (the prosodic transfer hypothesis), which is regarded as an opponent of the RDH in the study of Goad et al. (2003) and Goad and White (2006). In terms of the PTH, Chinese learners should have a similar suppliance performance of the morpheme ‘*-s*’ in the spoken production, as ‘*-s*’ for plural nouns and 3SG thematic verbs are prosodically identical in English. Nevertheless, from the view of the oral data in this study, the plural ‘*-s*’ (75.9%) was inflected considerably more than 3SG ‘*-s*’ (29.4%) with Sig = 0.000 < 0.05. In this case, the PTH fails to account for the different scores in supplying the plural ‘*-s*’ and 3SG ‘*-s*’ which share the same prosodic representation.

### The Exclusion of Irregular Markings and Inflections in Formulas

When focusing on the irregular plural markings, this research found that the suppliance rate was quite high in verbal inflections and in nominal inflections (but with little occurrences), which is distinguished from the low suppliance rate of regular affixations. Table 9 shows the comparison between the regular and irregular markings for verbs and nouns from the two tasks, respectively. In Table 9, all auxiliary verbs were counted as irregular markings. These divergent suppliance rates supported the idea that irregular markings occupied a separate area from regular markings in L2 learners’ minds (Marzilli and O’Brien, 2000; Hawkins and Liszka, 2003).

**Table 9:** Suppliance rates of regular and irregular markings for 3SG verbs and plurals.

	3SG verbal inflections		Plural nominal inflections	
	Regular	Irregular	Regular	Irregular
Spoken data	29.4% (78/265)	96.9% (471/486)	75.9% (305/402)	100% (1/1)
Written data	74.1% (347/468)	100% (109/109)	91.35% (317/347)	92% (23/25)

On the other hand, 3SG and plural markings in formulaic expressions in spoken task also indicated that it was a disparate way to make morphological inflections when the inflections were in non-formulas, as presented in Table 10. Abney (1991) holds the view that the co-occurrence of chunks is rarely generated by its syntactic representations. The highly frequent inflected form in formulaic language is supposed to be obtained by a fixed concept (Penke, 2012). Although both irregular and regular inflections have the same syntactic feature (3SG or PL), the different inflectional morphology may be achieved through different processes. Hence, when investigating L2 competence in inflectional morphology, it is paramount to distinguish the target features from the chunk structure.

**Table 10:** Suppliance rates of 3SG auxiliary verbs and regular plurals (± chunk).

	Aux. V with pronouns	Aux. V without pronouns	Plural in chunk	Plural not in chunk
Spoken data	99.4% (311/313)	93.2% (126/133)	75.9% (305/402)	90% (9/10)

## Conclusion

The study provided practical evidence for the RDH by investigating thirty-three Chinese-speaking adults’ verbal and nominal inflections with two different research paradigms. The main attention was placed on the inflectional morphology in the obligatory contexts of 3SG and plurals. First, plural ‘*-s*’ was marked more than 3SG ‘*-s*’ both in the conversation task and in the written translation task. This may result from the fact that a plural morpheme ‘*-men*’ in L1 Chinese can facilitate the acquisition of plural ‘*-s*’ in L2 English, as implied in the RDH. In contrast, due to the lack of subject–verb agreement markings in L1, the participants had difficulty in establishing the connection between syntactic feature of 3SG and the corresponding morphological inflections, and thus more omission was found for 3SG ‘*-s*’. Second, the majority of the participants were exposed to English at the mean age of ten years old, which is during late childhood. However, the RDH proposes that early childhood is the pivotal stage to acquire the L2 morphosyntactic feature that is absent in L1, and once learners miss that stage, there will be a permanent impairment to acquire the feature. Third, some exceptional cases where the plurals were inflected less than 3SG were found exclusively in the written task. Nonetheless, this phenomenon was not regarded as an evidence against the RDH. Instead, it means that the way the participants marked 3SG ‘*-s*’ was dependent on the output checking system rather than the interface between lexicon and morphophonology. Accordingly, the researchers speculated that as the participants could not develop the correlation between 3SG feature and affixation marking, they needed to ask for help from the output checking system which is more active in a written context than in a spoken context to increase the inflectional rates. Fourth, the overgeneralization of affix ‘*-s*’ was more common in plural markings, which may be attributed to the better acquisition of plural affixal markings. Besides, a worth mentioning finding is the high inflection rates for irregular markings, and for the regular markings in a chunk. It indicated that the way the participants produced these two inflections was dissociated with the construction of the regular markings without a chunk. and thus it should be analyzed especially.

Based on all findings and discussion, there are some implications for the pedagogy and further research: (1) Chinese students show optionality in marking 3SG and plural inflections. To increase L2 accuracy, more attention should be paid when imparting the corresponding knowledge. First, students should learn English as a second language at an earlier age to compensate for the impairment of 3SG inflections predicted in the RDH. Second, even if learners have missed the critical period for acquiring the morphosyntactic features, we can intentionally increase input frequency to raise the awareness of inflectional morphology. (2) it is suggested to isolate the ‘chunk’ expressions to increase experimental validity when investigating 3SG and plural markings, or other inflections.

Finally, there are some limitations in the study. First, we cannot exclude the potential effect of the proficiency level. Even though plural ‘*-s*’ outperformed 3SG ‘*-s*’ for the two tasks, the participants were supposed to be at an upper-intermediate proficiency level with an overall IELTS ranged from 5.5 and 6.5. Therefore, we do not know whether the results will still be consistent with an advanced group, whose IELTS score may be over 7. Second, the influences from acquisition order and input frequency on L2 morphological acquisition cannot be excluded. Third, this research did not answer why the inflection markings of plural *‘-s’* did not reach a native-speaker level, even though it behaved better than 3SG ‘*-s*’. I speculate that it may be caused by the limited application range for ‘*men*’ in Chinese, which must follow a noun with a human property and is incompatible with a numeral. To test whether the limited applicability of ‘*men*’, further research is recommended to investigate the suppliance rate of marking plural ‘*-s*’ for human nouns, and when connecting with quantifiers.

## Data Availability Statement

The raw data supporting the conclusions of this article will be made available by the authors, without undue reservation.

## Ethics Statement

The studies involving human participants were reviewed and approved by University of Sheffield. The patients/participants provided their written informed consent to participate in this study.

## Author Contributions

NL raise the idea of the study and contributed to the data collection, data analysis, and manuscript drafting. LY contributes to the research design and manuscript revision. All authors contributed to the article and approved the submitted version.

## Funding

This research was funded by the Chinese National Social Science Fund Key Project “Chinese EFL Learners’ Second Language Pragmatic Competence Research” (17AYY023).

## Conflict of Interest

The authors declare that the research was conducted in the absence of any commercial or financial relationships that could be construed as a potential conflict of interest.

## Publisher’s Note

All claims expressed in this article are solely those of the authors and do not necessarily represent those of their affiliated organizations, or those of the publisher, the editors and the reviewers. Any product that may be evaluated in this article, or claim that may be made by its manufacturer, is not guaranteed or endorsed by the publisher.

## Supplementary Material

The Supplementary Material for this article can be found online at: https://www.frontiersin.org/articles/10.3389/fpsyg.2022.930504/full#supplementary-material

## Footnotes

^1^According to Lin (2015), past tense may exist in Chinese, but not under the same criteria as the English tense system. For example, past tense in Chinese is pronominal, while requires a morphological inflection for verbs in English.

^2^‘LE’ is one of perfective aspect markers in Chinese (Klein et al., 2000).

^3^According to Cohen (1988), *η*^2^ = t^2/ (t^2+ (n-1)), where t = T-test statistic value, and *n* = the number of paired cases. If *η*^2^ around 0.01, there is a small effect; If *η*^2^ around 0.06, there is a median effect; If *η*^2^ around 0.14, there is a large effect.

^4^The suppliance rates of marking 3SG copula *‘be*’ verb when connecting with pronouns and without pronouns did not coincide with normal distribution. Hence, Bland and Altman (1995) suggest that the non-parametric test: Wilcoxon signed-rank test, which is equivalent to paired samples t-test, should be conducted for this case.

^5^It was found that some participants (like No.01) translated ‘linshi’/‘snacks’ to ‘snakes’. In this case, although ‘snakes’ was not correct from the aspect of translation, it was counted as a plural marking, as this study focused on whether the students have the ability to add the morpheme ‘-*s*’ for regular countable nouns to interpret the plural feature. The production of ‘snakes’ may imply that the participants were able to add affix ‘-*s*’ to a bare noun to express a plural feature.

## Abbreviations

3SG: Third person singular
Aux. V.: Auxiliary verbs
FFFH: Failed functional features hypothesis
FL: Formulaic language
IL: Interlanguage
L1: First language
L2: Second language
RDH: Representational deficit hypothesis
S-V-O: Subject-verb-object
T: Tense
