# Supplementary material for: Testing the Representational Deficit Hypothesis: From the Aspect of Chinese Learners’ Acquisition of Affixation ‘-s’ for Third Person Singular Verbs and Plural Nouns
Source: Front Psychol. 2022 Jun 10;13:930504. doi: 10.3389/fpsyg.2022.930504 (PMC9231562; doi:10.3389/fpsyg.2022.930504)
Supplement: Supplementary file 1 [file Data_Sheet_1.PDF]

Appendix 1

Personal Details

1. Date of birth: __ /____ (month/year)
2. Native language: _________
3. How long have you lived in an English speaking country/countries?

   ____Less than one month

   ____One month to half a year

   ____Half a year to one year

   ____More than one year
4. How many years have you studied English as a second language?

   _____ year(s)
5. What is the score of your latest IELTS test?

   Listening: ___ Reading: ___ Speaking: ___ Writing: ___

Overall: ___

6. When did you get this score?

   __/__/____ (day/month/year)
7. If you have any more information about your background that is relevant to the language, please explain:

____

____
