# Supplementary material for: Testing the Representational Deficit Hypothesis: From the Aspect of Chinese Learners’ Acquisition of Affixation ‘-s’ for Third Person Singular Verbs and Plural Nouns
Source: Front Psychol. 2022 Jun 10;13:930504. doi: 10.3389/fpsyg.2022.930504 (PMC9231562; doi:10.3389/fpsyg.2022.930504)
Supplement: Supplementary file 2 [file Data_Sheet_2.PDF]

Appendix 2

Spoken task (a 5-10-minute conversation)

1. Can you describe your mother?
2. How old is she?
3. What is her job?
4. What does she usually do on weekdays?
5. What does she usually do on weekends?
6. Can you tell me your mother's hobbies?

7. Have you found a good friend here?
8. Who is your best friend?
9. What does she/he usually do?
10. What are her/his hobbies?

11. Who is your favourite star? Like a singer or a movie star?
12. Why do you like him/her?
13. What is her/his personality?

14. Who is your favourite teacher in China/the UK?
15. Why do you like her/him?
16. What is her/his personality?
17. What about your teacher in your present class? Your 10-week pre-session class in a foreign country?
18. Can you tell any difference between the teacher in China and in the UK?

19. Do you have a pet?

20. What is it? Is it a dog or a cat？

21. What does she/he usually eat / play?
22. What does she/he usually do when she/he stays with you?
23. Does your mother like your pet?
