# Supplementary material for: Testing the Representational Deficit Hypothesis: From the Aspect of Chinese Learners’ Acquisition of Affixation ‘-s’ for Third Person Singular Verbs and Plural Nouns
Source: Front Psychol. 2022 Jun 10;13:930504. doi: 10.3389/fpsyg.2022.930504 (PMC9231562; doi:10.3389/fpsyg.2022.930504)
Supplement: Supplementary file 3 [file Data_Sheet_3.PDF]

**Appendix 3**

**Written task**

1. Please translate the following text into English. (Topic: my mother's daily routine, which means that those activities happen every day)

我的妈妈通常在六点钟起床。然后她去刷牙洗脸。我家养了两只宠物。一只名叫 Henry 的狗和一只名叫 Kitty 的猫。我的妈妈在早晨六点半喂它们。之后，她开始准备早餐。早餐通常有三个鸡蛋，一些新鲜的水果和三杯牛奶。吃完早餐后，我爸爸去上班，我去上学了。然后我妈妈会扫下地。 十点左右，她出去买接下来几天所需要的食物，比如零食，水果，饼干等等。中午的时候，她开始做中饭并且喂两只宠物。下午时，她和她的朋友出去购物或者在图书馆读书。如果外面下雨的话，她一般会呆在家里看电视。在准备晚餐之前，她把狗带到附近的街道去遛一遛，而 Kitty 则被锁在家里。因为它是一只猫咪。我的爸爸在晚饭之后洗碗。我的妈妈在九点左右洗澡。她经常在睡前给我讲故事。她说这样有助于我有一个好的睡眠。

_______________________________________________

_______________________________________________

_______________________________________________

_______________________________________________

_______________________________________________

_______________________________________________

_______________________________________________

_______________________________________________

_________________________________
