# Supplementary material for: Testing the Representational Deficit Hypothesis: From the Aspect of Chinese Learners’ Acquisition of Affixation ‘-s’ for Third Person Singular Verbs and Plural Nouns
Source: Front Psychol. 2022 Jun 10;13:930504. doi: 10.3389/fpsyg.2022.930504 (PMC9231562; doi:10.3389/fpsyg.2022.930504)
Supplement: Supplementary file 4 [file Data_Sheet_4.PDF]

Appendix 4

Transcription for the written task

My mother always gets up at 6 am. And then she brushes her teeth and washes her face. We keep two pets. One dog is called Henry and one cat is called Kitty. My mother feeds them at 6.30 am. After that, she starts to prepare the breakfast. The breakfast always contains 3 eggs, some fresh fruit and three cups of milk. After eating breakfast, my father goes to work and I go to school. Then, my mother sweeps the floor. Later, she goes out to buy some food for the following days, such as snacks, vegetables, cookies and so on. In the midday, she cooks lunch and feeds two pets. In the afternoon, she goes shopping with her friends or reads books in a library. If it rains, she will watch TV at home. Before she prepares the dinner, she walks the dog around local streets, while Kitty is locked at home because Kitty is a cat. After dinner, my father washes dishes. And my mother takes a shower at 9 pm. She always reads stories for me before sleeping. She says that it is good for me to have a nice sleep.
