# Supplementary material for: Testing the Representational Deficit Hypothesis: From the Aspect of Chinese Learners’ Acquisition of Affixation ‘-s’ for Third Person Singular Verbs and Plural Nouns
Source: Front Psychol. 2022 Jun 10;13:930504. doi: 10.3389/fpsyg.2022.930504 (PMC9231562; doi:10.3389/fpsyg.2022.930504)
Supplement: Supplementary file 5 [file Data_Sheet_5.PDF]

Appendix 5

Information Sheet

1. What is the research about?

The survey I will ask you to complete is part of a project that tests the representational deficit hypothesis.

2. Who can participate?

Participants should meet two requirements: (1) Chinese learners of English whose age is above 18; (2) the latest overall score of IELTS is between 5.5 and 6.5, which correspondents to B2 level in CEFR (Common European Framework of Reference).

3. What does this research involve?

There are two tasks in this research. As for the spoken task, participants need to have a 5-10 minute conversation with the experimenter. And then there is a 2-minute break. After the break, the written task will be conducted. Participants need to translate the Chinese text into English, which contains about 120 words.

4. Do I have to take part?

No, participation is entirely voluntary. You are free to quit at any time before the end of the survey, and your data will be destroyed and will not be used in the research.

5. Are there any risks to taking part?

We are not aware of any risks to taking part.

6. What will happen to my responses to the survey?

Your responses will be used alongside the responses of other participants, and will be stored securely in the University of Sheffield, School of English.

7. What about confidentiality?

The task responses are analysed anonymously, and therefore there will be no record that identifies you with the responses you give. No participant information will be used in any presentation or related to the research.

8. Will I know the results?

If you would like to receive a summary of the group results of the two tasks, please send me an email to the address below and I will email the summary to you.

Ni Li: liniemai@163.com
